# A reproducible pipeline for TCR β-chain repertoire analysis from thoracic tumour RNA-seq and ICI-associated cardiac immunotoxicity

**DOI:** 10.3389/fimmu.2026.1892720

**Published:** 2026-09-02

**Authors:** Xu Liu, Gaojun Xu, Shuaifei Wang, Shiquan Zhang, Wentao Wu, Zhenwei Ge

**Affiliations:** Department of Cardiovascular Surgery, Henan Provincial Chest Hospital, Zhengzhou, China

**Keywords:** bulk RNA-seq, bystander T cells, cardio-oncology, clonality, cohort-level quality control, cross-compartment TCR sharing, immune checkpoint inhibitor, immune-related adverse events

## Abstract

**Background:**

Bulk RNA-sequencing of thoracic tumours is routine, but the T-cell receptor (TCR) repertoires it contains are rarely re-mined with a uniform pipeline, and library-prep compatibility for productive V–J-spanning assembly is rarely audited at the cohort level for non-small-cell lung cancer (NSCLC) and oesophageal squamous-cell carcinoma (ESCC).

**Objective:**

We built a reproducible, openly-released pipeline for productive T-cell-receptor β-chain (TRB) repertoire reconstruction from public thoracic-tumour RNA-seq, and extended it to immune-checkpoint-inhibitor (ICI)-associated cardiac immunotoxicity.

**Methods:**

Four GEO cohorts (NSCLC and ESCC bulk RNA-seq) were assembled with TRUST4 under a transparent cohort-level QC rule (≥ 10% V+J assemblies on ≥ 1 representative sample), and antigens were annotated by exact CDR3-amino-acid matching to VDJdb. The identical pipeline was applied to 23 peripheral-blood TRB repertoires from an ICI-irAE single-cell cohort (GSE180045; 10 discovery and 13 validation libraries).

**Results:**

The retained pilot dataset comprised 12 productive samples and 916 productive TRB clones; ESCC (n = 1 after QC) was oligoclonal versus diverse NSCLC. Of 37 VDJdb hits, 33 were viral and 4 tumour or self, with no clone shared across cohorts. Thoracic-tumour tissue repertoires were significantly more oligoclonal than peripheral ICI-irAE repertoires (top-10 clone fraction 0.76 vs 0.18; inverse Simpson 8.8 vs 177; Mann-Whitney BH-FDR < 0.001), whereas depth-normalised metrics did not differ (FDR = 0.61), indicating an assay-platform (bulk RNA-seq versus 10x single-cell), sequencing-depth and tissue-versus-blood effect rather than intrinsic clonal architecture; within-patient cardiac contrasts were not significant and are reported descriptively.

**Conclusion:**

One uniform pipeline transparently re-mines public thoracic-tumour and cardiac-immunotoxicity RNA-seq for TCR repertoires. These findings are descriptive and hypothesis-generating rather than statistically generalisable, with the ESCC-versus-NSCLC contrast resting on a single ESCC sample.

## Introduction

Thoracic malignancies, chiefly non-small-cell lung cancer (NSCLC) and oesophageal squamous-cell carcinoma (ESCC), were among the first solid tumours in which programmed-death-1 (PD-1) blockade produced durable, registrational responses (1–4). T cells reactive to tumour-derived peptides drive these responses. The diversity, clonality and antigen specificity of intratumoural and peripheral T-cell receptor (TCR) repertoires have repeatedly emerged as candidate biomarkers of immune-checkpoint-inhibitor (ICI) outcome across solid tumours (5–10). Recent NSCLC trials have moved PD-1 blockade into the neoadjuvant setting (2), and similar combinations are now approved in resected oesophageal and gastro-oesophageal-junction cancer (11). Reviews of the thoracic immune microenvironment emphasise the heterogeneity of these repertoires and the need for atlas-scale resources that can be re-interrogated with new tools (12, 13).

The same PD-1/CTLA-4 blockade that drives these thoracic-tumour responses can also trigger immune-related adverse events, of which immune-checkpoint-inhibitor (ICI)-associated myocarditis is among the most feared: although it occurs in roughly 1% of treated patients, reported fatality reaches up to ~50% (14–16). Pharmacovigilance and single-centre series have established its clinical severity (16, 17), and single-cell studies of affected patients have shown that cytotoxic CD8+ T cells undergo clonal expansion in myocarditis, implicating a shared, antigen-driven T-cell mechanism that may overlap with anti-tumour immunity (18). Whether the T-cell receptor repertoires engaged in thoracic tumours and in ICI cardiotoxicity can be read with a single, uniform pipeline — and how their diversity, clonality, antigen specificity and clonal sharing compare — has not been examined. Because the same checkpoint biology and the same bulk- and single-cell sequencing substrates underlie both settings, a cross-compartment TCR view is both feasible and clinically motivated.

Direct TCR-sequencing on sorted T cells remains the most sensitive way to read these repertoires (19, 20). It is rarely co-deposited with the bulk RNA-seq that is now routine for thoracic-tumour cohorts. Several assembly-based methods now address this. MiTCR (19), MiXCR (21), V’DJer (22), TRUST (23), CATT (24), ImReP (25) and TRUST4 (26) reconstruct productive CDR3 rearrangements from the small fraction of TCR-spanning reads in bulk RNA-seq. Head-to-head benchmarks are clear that method sensitivity varies sharply with library-prep chemistry, read length and the T-cell fraction of total RNA (20, 27, 28). Cohort-level surveys of immunoglobulin and TCR repertoires across public RNA-seq have demonstrated the scale that is now reachable (23, 25). The field has accumulated curated antigen-specificity resources, including VDJdb (29), McPAS-TCR (30), IEDB (31) and TCRdb (32). HLA-typing tools that run on the same RNA-seq input, such as arcasHLA (33), OptiType (34) and HLA*LA (35), are also available.

Despite this maturity, three practical gaps remain when bulk RNA-seq is used as a primary substrate for TCR profiling at cohort scale. First, individual samples are routinely audited for sequencing depth and contamination. Cohort-level assembly quality control — whether a library prep yields V- and J-spanning contigs at all — is rarely tested before samples are pooled into a downstream analysis. Second, the antigen-recognition layer is often a peripheral annotation, even though it is exactly what links a CDR3 amino-acid string to a biological hypothesis (36–39). Third, single-cell TCR atlases of pan-cancer T cells (40, 41) and oesophageal-tumour ecosystems (42, 43) now exist at high resolution. The much larger inventory of bulk thoracic RNA-seq has not been re-mined with a consistent, openly-released pipeline that surfaces the same diversity, clonality, V/J-usage and antigen-database hits.

**Figure 1:**
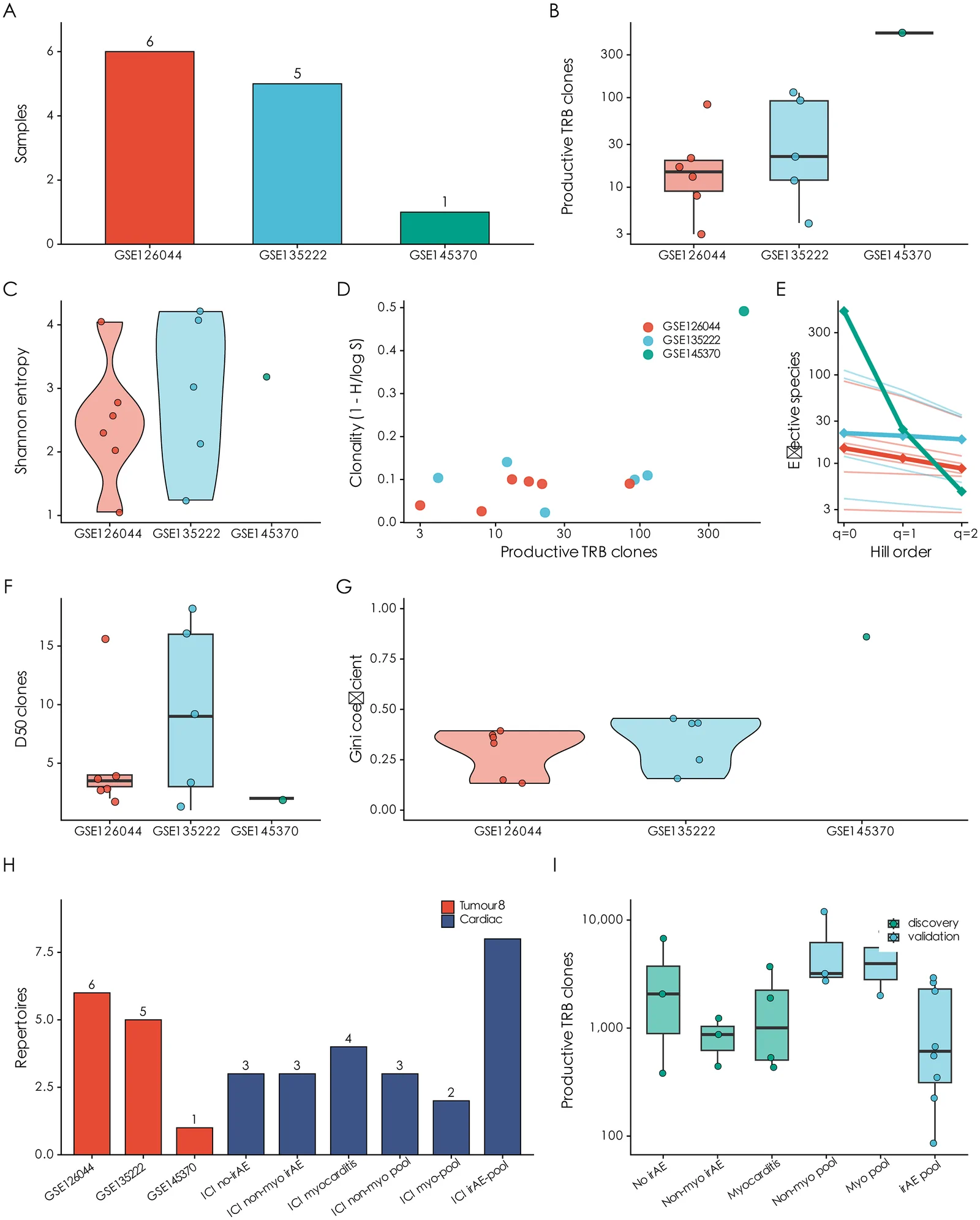
Cohort composition and clone-count landscape of the retained pilot dataset. **(A)** Number of productive samples retained per cohort after the cohort-level V+J-assembly QC rule and fastq integrity QC (n = 6 GSE126044, n = 5 GSE135222, n = 1 GSE145370). **(B)** Per-sample productive TRB clone counts by cohort, displayed as overlaid jitter on box plots; y-axis on a log scale. **(C)** Shannon entropy of the read-count-weighted productive-TRB distribution per sample, displayed as violins with overlaid jitter. **(D)** Clonality (1 − Shannon/log S) plotted against productive-TRB count per sample. **(E)** Hill-number profiles per sample for q ∈ {0, 1, 2}; per-cohort medians plotted as solid lines. **(F)** D50 (number of clones contributing the top 50% of reads) by cohort. **(G)** Gini coefficient of the read-count distribution by cohort. Colour scheme: NPG palette throughout; cohort colour key in each panel. **(H)** Extended atlas composition including the cardiac ICI-irAE cohort (GSE180045; discovery and validation batches): the three thoracic-tumour cohorts and the cardiac groups, coloured by compartment (tumour tissue versus peripheral blood), shown as a landscape inventory only. **(I)** Per-library clonal depth of the cardiac repertoires by ICI disease group, with the discovery and validation cohorts coloured separately. Panels **(H, I)** are descriptive additions to the atlas landscape and carry no significance claims.

**Figure 2:**
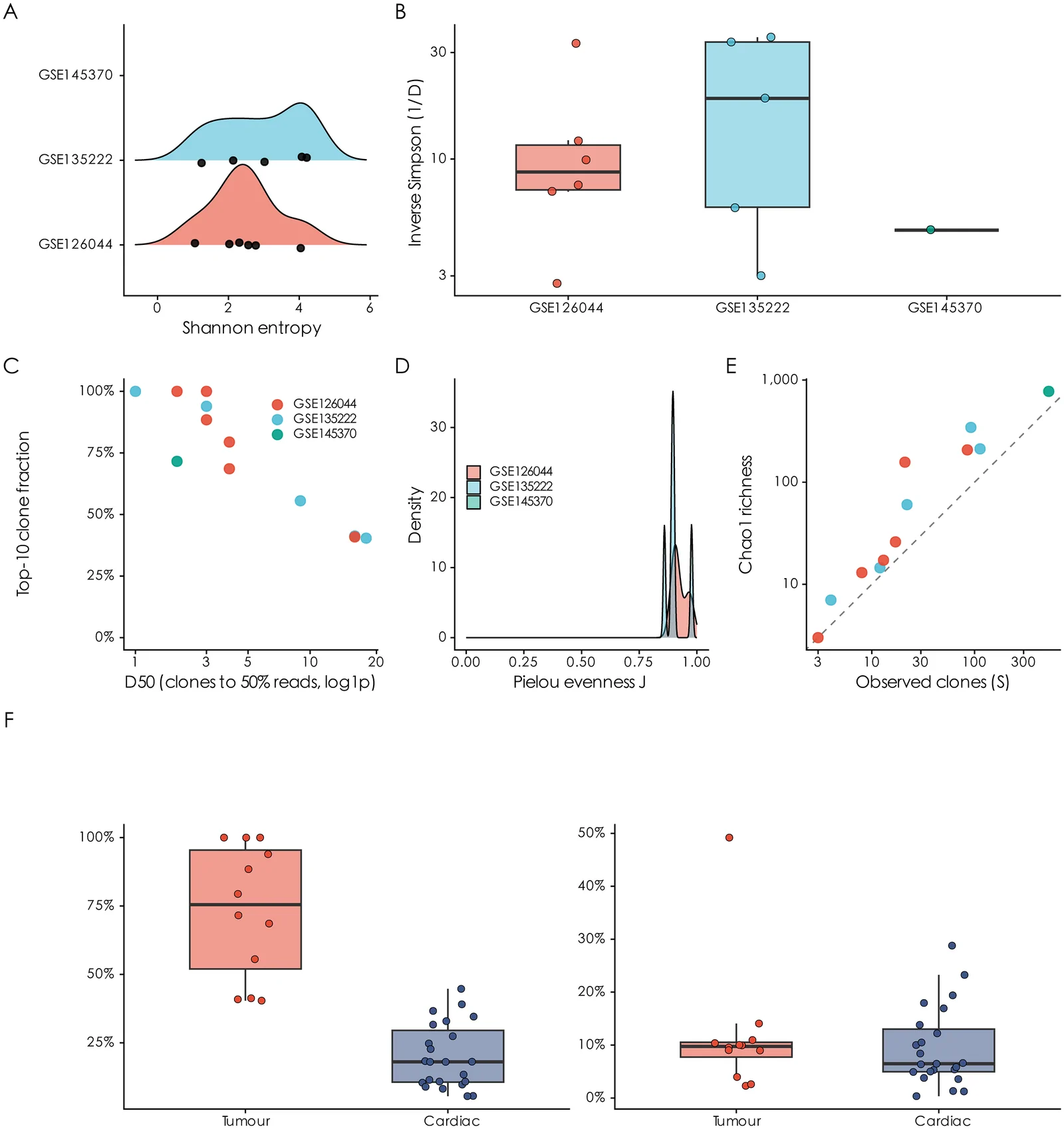
Diversity and clonality metrics. **(A)** Per-sample Shannon entropy displayed as cohort-level ridge plots with overlaid jitter. **(B)** Inverse Simpson index (1/D) per sample by cohort, on a log scale. **(C)** Per-sample top-10 clone fraction (cumulative read share of the top-10 clones) plotted against D50 on log1p scale. **(D)** Pielou evenness density distributions per cohort. **(E)** Chao1 richness versus observed clone count on a log–log scale; dashed identity line. **(F)** Tumour-versus-cardiac compartment comparison of clonal structure (cardiac repertoires from GSE180045; **Supplementary Table 8**). Left: the only statistically significant contrast — the top-10 clone fraction is higher in thoracic-tumour tissue repertoires than in peripheral ICI-irAE repertoires (Mann-Whitney, Benjamini-Hochberg FDR < 0.001). Right: depth-normalised clonality does not differ between the two compartments (n.s., FDR = 0.61). The subtitle notes the sequencing-depth and tissue-versus-blood confound.

Here, we close part of that gap with a reproducible end-to-end pipeline. The pipeline begins at the European Nucleotide Archive (44) and Sequence Read Archive (45) record level. It assembles productive TRB clones with TRUST4 (26) and applies a transparent cohort-level V+J-assembly QC rule that excludes libraries on which the inference is unreliable. Standard diversity (46–50), clonality and CDR3-motif features are then derived, and antigen specificity is annotated by exact CDR3-amino-acid matching to VDJdb (29). We apply the pipeline to four public thoracic-tumour RNA-seq cohorts (42, 51–53), three NSCLC anti-PD-1 series and one paired-treatment ESCC series. The pilot atlas contains 12 productive repertoires, 916 productive TRB clones and 37 VDJdb-annotated antigen hits across NSCLC and ESCC. The pipeline, atlas object and per-figure scripts are released as a starting resource that future studies can extend cohort-by-cohort, including those with controlled-access TCGA data and integrated motif clustering (54–56). We then extend the same pipeline to an ICI-irAE single-cell cohort, adding 23 peripheral-blood cardiac repertoires to the atlas landscape (**Figures 1H, I**) and a direct tumour-versus-cardiac comparison (**Figure 2F**; **Supplementary Table 8**).

## Methods

### Cohort discovery and manifest assembly

Public bulk RNA-seq runs were queried programmatically from the European Nucleotide Archive (ENA) (44) and resolved through NCBI E-utilities for GEO-to-SRP mapping against the Sequence Read Archive (45). Eligible runs were filtered to library_strategy = RNA-Seq, library_layout = PAIRED, and Illumina platforms (HiSeq 2500, NovaSeq 6000). Four GEO cohorts matched the thoracic-tumour scope: GSE126044 (51) and GSE135222 (52) (non-small-cell lung cancer, anti-PD-1 nivolumab), GSE145370 (42) (oesophageal squamous-cell carcinoma) and GSE193258 (53) (NSCLC neoadjuvant pembrolizumab). The combined ENA manifest contained 121 paired-end RNA-seq runs totalling ~1 TB of fastq. For the pilot atlas, runs within each cohort were ranked by published productivity proxies and capped at 25 samples (configs/pilot_geo_10.csv, configs/atlas_geo_25.csv).

### fastq download and integrity QC

fastq files were retrieved from the ENA HTTP mirror using aria2c v1.37.0 with adaptive concurrency (--max-connection-per-server=8, --split=8) and per-file gzip-integrity verification (gzip -t). Runs whose R1 or R2 returned PARTIAL or MISSING after a re-download attempt were marked as fastq-incomplete and excluded from assembly (**Supplementary Table 4**). After this step, the runs passing integrity QC entered TRUST4; the complete per-step attrition (25 selected runs → fastq-integrity exclusions → cohort-level QC exclusion of GSE193258 → productive-TRB filter → 12 retained productive samples) is detailed in **Supplementary Table 1** and **Supplementary Figure S8**.

### TCR repertoire reconstruction

Reads were processed with TRUST4 v1.1.5-r573 (26) using the IMGT-derived reference hg38_bcrtcr.fa and human_IMGT+C.fa. TRUST4 was run in paired-fastq mode (run-trust4 -f hg38_bcrtcr.fa --ref human_IMGT+C.fa -1 R1.fastq.gz -2 R2.fastq.gz) on each sample. Output *_report.tsv and *_airr.tsv were parsed; only contigs flagged productive = T, with a complete TRBV–CDR3-amino-acid–TRBJ rearrangement and an in-frame CDR3-aa length ≥ 6, were retained. Productive-TRB clones with identical CDR3-aa + V + J were collapsed and read counts summed per sample. TRUST4 also reports TRA contigs; for this release all downstream analyses were restricted to the TRB chain, and TRA repertoires are not reported here.

### Cohort-level assembly QC

Library-prep chemistry and read length can systematically suppress V–J-spanning CDR3 assemblies in bulk-RNA-seq TCR inference (20, 27, 28). To avoid pooling samples from such libraries, we evaluated the fraction of TRUST4 contigs containing both a V-gene and a J-gene call per representative sample. A cohort-level inclusion rule was set at ≥ 10% V+J assemblies on ≥ 1 representative sample. The 10% cutoff was chosen pragmatically, as an order-of-magnitude separation between the V+J-rich retained cohorts (70.5–83.1% V+J) and the failing cohort (0.0–5.3% V+J), rather than as a finely calibrated value; evaluating at least one representative sample per cohort was considered sufficient because the library-prep chemistry that drives V+J recovery is shared within a cohort. GSE193258 failed this rule at 0.0–5.3% V+J on the two samples that completed assembly (**Supplementary Table 6**) and was excluded from the atlas before downstream analysis. CDR3-nucleotide-length distributions and V-only/J-only/V+J/none assembly fractions were tabulated for documentation (**Supplementary Figure S4**).

### Diversity and clonality metrics

Per-sample repertoire metrics were computed in Python on the read-count-weighted productive-TRB-clone vector $n=(n_{1},\ldots ,n_{S})$ with $p_{i=n_{i}/\sum_{j}n_{j}}$:

- Shannon entropy (46): $H=-\sum_{i}p_{i}\log p_{i}$ (natural log).
- Simpson index (49): $D=\sum_{i}p_{i}^{2}$; inverse Simpson 1/D.
- Pielou evenness (50): J=H/logS.
- Gini coefficient on the sorted clone-frequency vector.
- Chao1 richness (48) with the Chiu correction; observed clones reported alongside.
- Hill numbers Dq for q∈{0,1,2} (47) with D1=exp(H) and D2=1/D
- Clonality: 1−H/logS.
- D50: the smallest number of clones whose cumulative read share exceeds 50%. (a lower D50 indicates a more dominated repertoire)
- Top-1, top-10 and top-100 cumulative read fractions.

Full per-sample values are in **Supplementary Table 2**. Rarefaction curves sampled reads at 10 evenly spaced fractions of total reads with 5 bootstrap replicates per fraction, plotting mean unique-clone counts with bootstrap range (**Supplementary Figure S6**).

### V/J usage and CDR3 motif analysis

**Figure 3:**
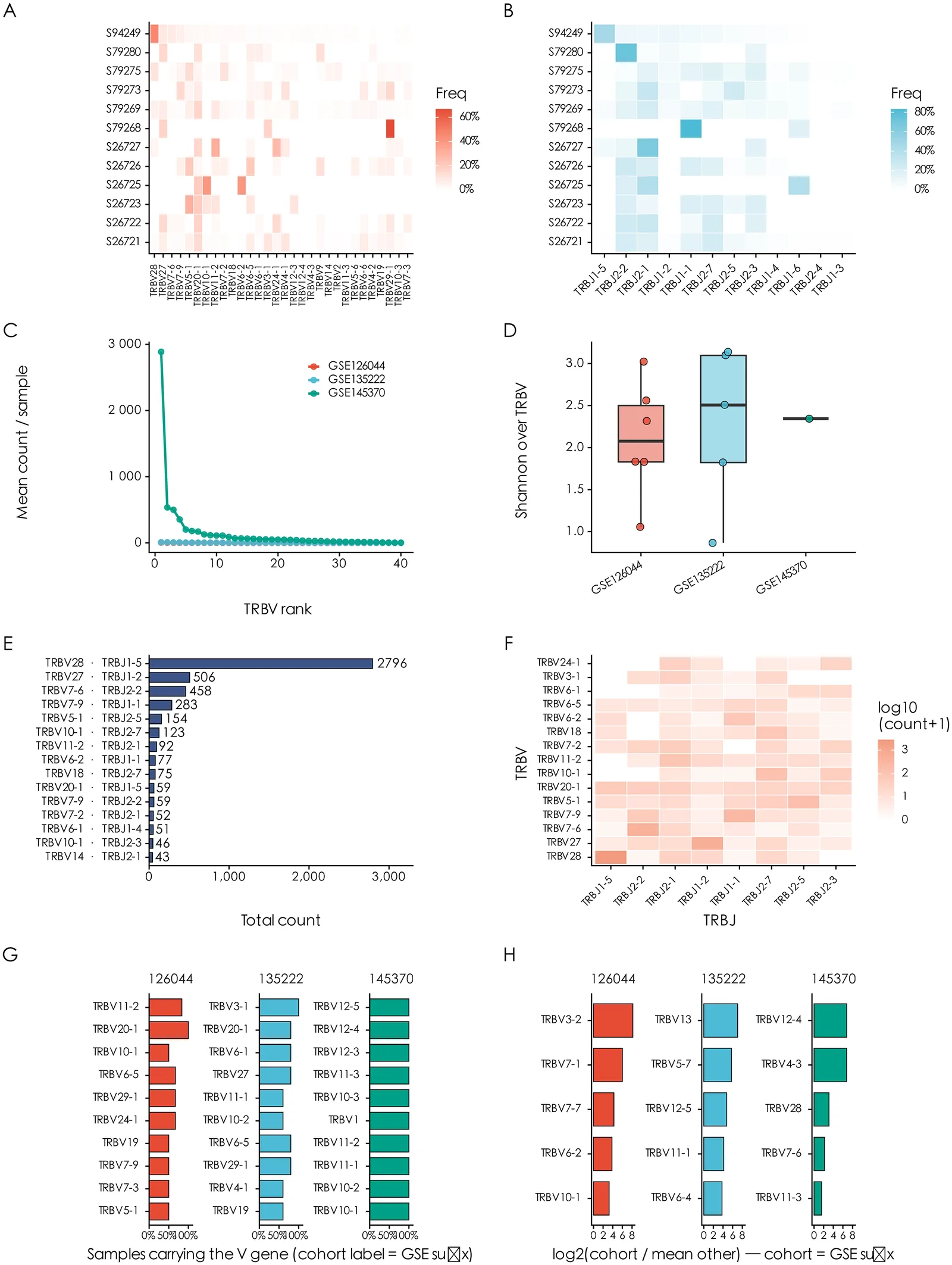
V/J-gene usage and TRBV–TRBJ pair landscape. **(A)** TRBV usage heatmap (per-sample TRBV frequencies) for GSE126044 + GSE135222 samples (NSCLC anti–PD-1). **(B)** TRBJ usage heatmap for the same samples. **(C)** Mean per-sample TRBV count by rank, coloured by cohort. **(D)** Per-sample Shannon entropy over TRBV usage by cohort. **(E)** Total reads per top-ranked TRBV–TRBJ pair across the atlas (top pair TRBV28–TRBJ1-5, 2,794 reads, contributed by the ESCC sample SRR11094249). **(F)** TRBV × TRBJ pair-count heatmap on log10(count + 1) scale. **(G, H)** Cohort-distinctive TRBV genes, ranked by log2(cohort-mean/other-cohort-mean) with read-count pseudo-counts.

**Figure 4:**
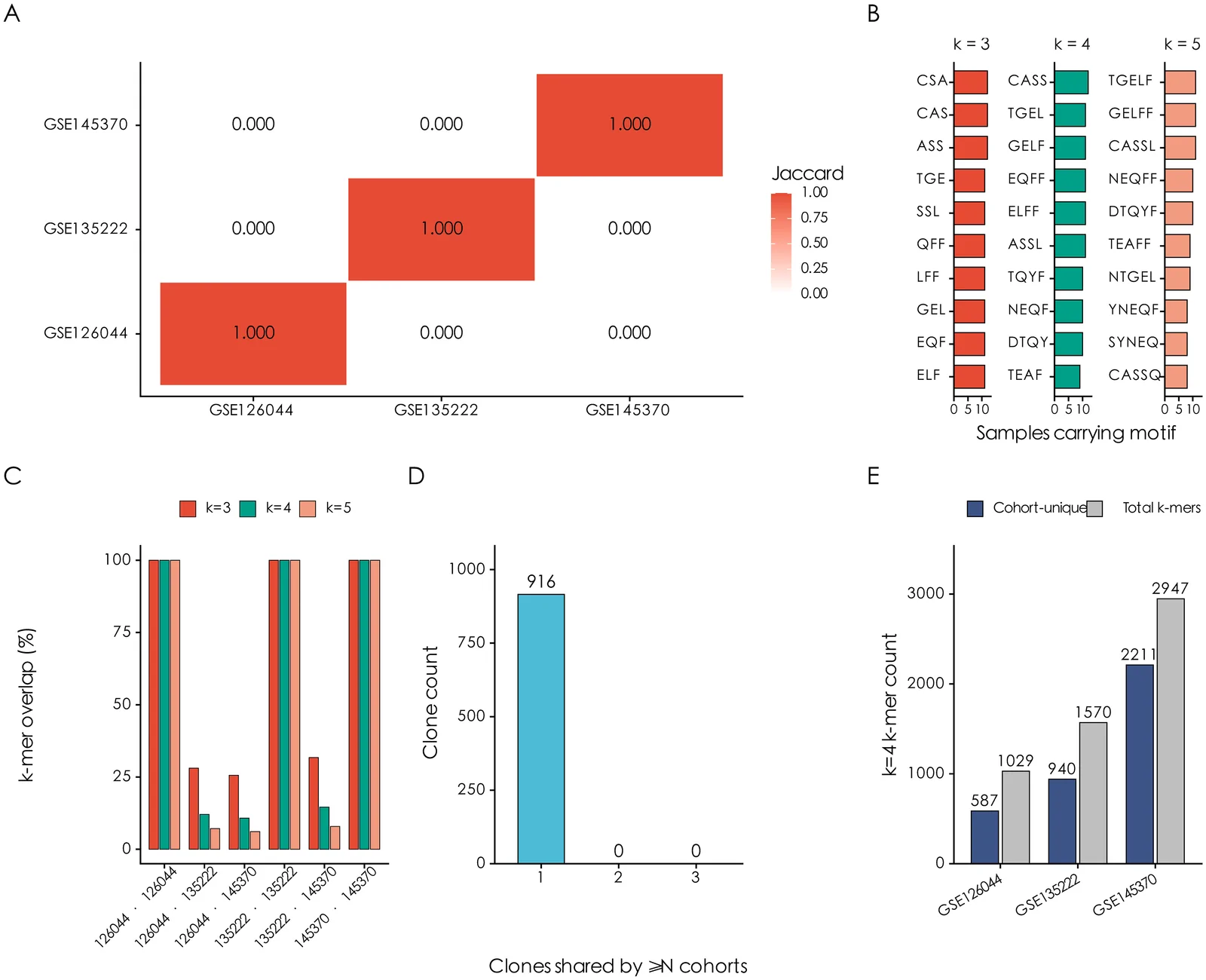
Cross-cohort CDR3 sharing and k-mer motif overlap. **(A)** Pairwise inter-cohort Jaccard similarity over full CDR3-amino-acid + V + J clonotypes (diagonal = 1.000 by definition; all off-diagonal values = 0.000). **(B)** Top k-mer motifs (k = 3, 4, 5) by number of samples carrying each motif. **(C)** Pairwise cohort-level k-mer-overlap percentages at k ∈ {3, 4, 5}. **(D)** Histogram of the number of cohorts each productive clone is carried by (916 clones in exactly one cohort; 0 in two; 0 in three). **(E)** Number of cohort-unique versus total k = 4 motifs per cohort.

TRBV and TRBJ counts per sample were aggregated and visualised as per-sample frequency heatmaps and cohort-level rank curves (**Figure 3**). Cross-cohort V-gene fold-changes were computed on log2(cohort-mean/other-cohort-mean) with a + 0.5 read pseudo-count. CDR3-amino-acid sequences were decomposed into overlapping k-mers (k ∈ {3, 4, 5}). For each k, we counted the number of samples and cohorts carrying each motif and the pairwise per-cohort k-mer Jaccard overlap (**Figure 4**). Cross-cohort clone-level Jaccard similarity was computed on the full CDR3-aa + V + J clonotype identifier.

### Public-database antigen lookup

**Figure 5:**
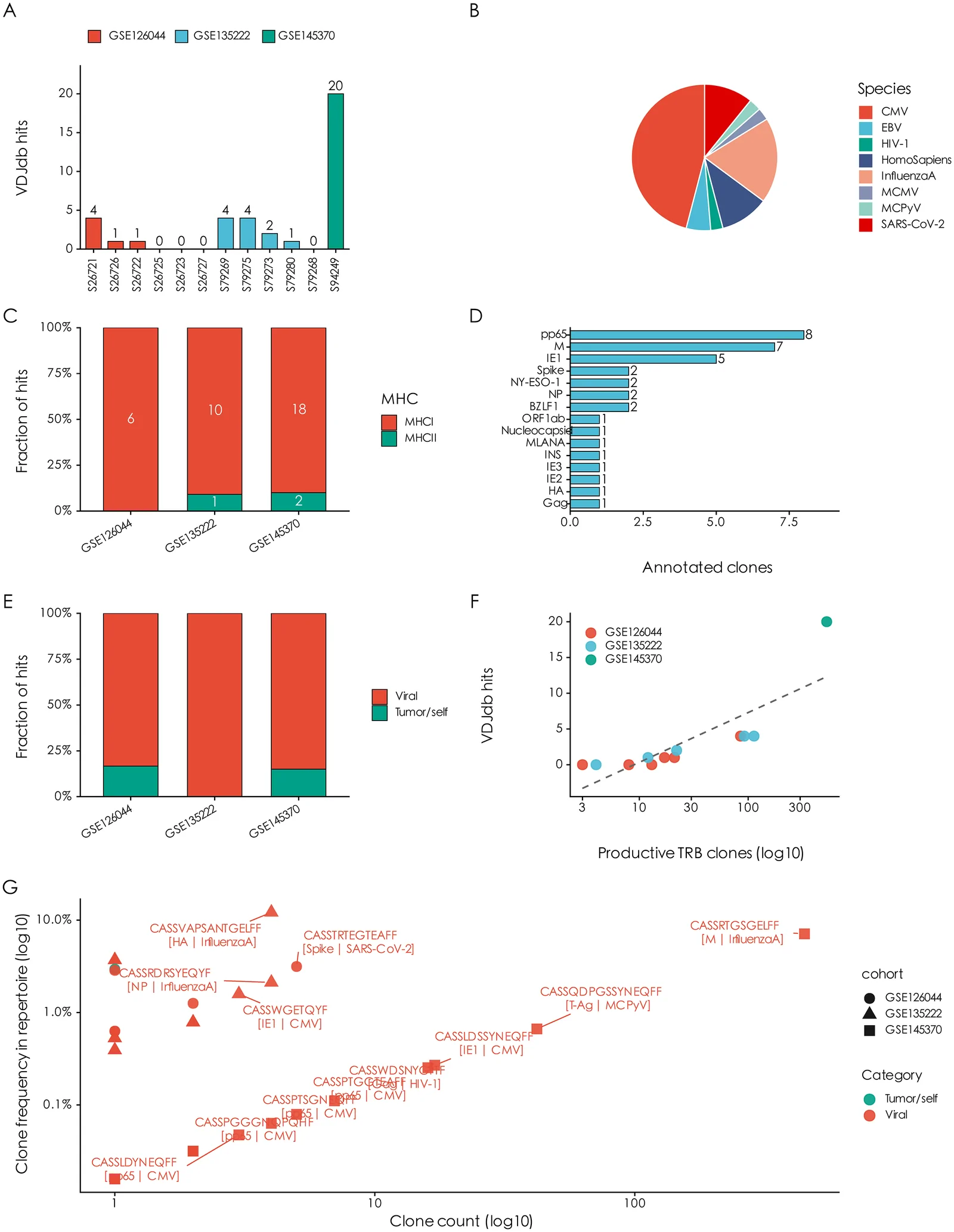
VDJdb-annotated antigen recognition. **(A)** Per-sample VDJdb hit count, coloured by cohort. **(B)** Pie chart of the species distribution across the 37 atlas-wide VDJdb hits. **(C)** Cohort-level MHC-class composition of VDJdb hits (MHC-I bars labelled with hit counts inside; MHC-II in the lower stack). **(D)** Top-10 antigen genes by annotated clone count atlas-wide. **(E)** Cohort-level breakdown of hits by category (viral vs tumour/self). **(F)** VDJdb hits per sample plotted against productive clone count on a log scale. **(G)** Per-sample top-frequency annotated clones with CDR3 sequence and (epitope ∣ species) labels.

We mirrored the VDJdb v2024.05 release (29) and indexed productive TRB clones against the database using exact CDR3-amino-acid matching. Lookups were restricted to entries with a non-empty epitope field and human MHC class I or II. McPAS-TCR (30) and IEDB (31) lookup modules were implemented but were not executed for this pilot release and are reserved for future expansion. Per-hit records (sample × clone × epitope × species × MHC class) are listed in **Supplementary Table 3** and visualised in **Figure 5** and **Supplementary Figure S7**. Tumour/self-versus viral categorisation followed VDJdb’s antigen.species and antigen.gene fields (Homo sapiens self-antigen genes — NY-ESO-1, MLANA, INS — versus all other species). For clarity, in this release only the TRUST4 assembly, diversity/clonality, V/J–CDR3 motif and exact-match VDJdb modules were executed; the McPAS-TCR, IEDB, arcasHLA, GLIPH2 and tcrdist3 modules are implemented but not run, and are reserved for future expansion.

### HLA typing

arcasHLA v0.6.0 (33) was used for paired-fastq HLA class-I/II four-digit typing from RNA-seq but was not executed on the current pilot atlas. Consequently, no HLA–TCR pairing analyses are reported here; this is acknowledged in the Limitations section.

### Statistical reporting and figure generation

All summary statistics in figures and the main text are descriptive (median, range, IQR) and accompanied by per-sample dot overlays where space permits, given the pilot sample size. No formal hypothesis tests are reported between the three thoracic-tumour cohorts, because GSE145370 contributed a single productive sample after QC, which precludes between-cohort inference with n = 1 in one arm; the only inferential tests reported in this study are the tumour-versus-cardiac compartment comparisons (Mann-Whitney U with Benjamini-Hochberg FDR), described in the Cardiac (ICI-irAE) repertoire subsection below. Figures were produced in R v4.4.2 using ggplot2 v4.0.1, ggsci v4.1.0 (NPG palette), patchwork v1.3.2, and supporting tidyverse packages (**Supplementary Table 5**), with R/00_setup.R defining a shared theme_thoracictcr() and palette_npg(). Each panel script is in R/fig*_*.R and corresponds 1-to-1 with the main and supplementary figures.

### Cardiac (ICI-irAE) repertoire extraction and statistics

Paired single-cell VDJ TCR-seq from an ICI-irAE cohort (GSE180045 (18)) was processed through the identical clonotype pipeline. CellRanger filtered-contig annotations were parsed; one productive TRB per cell barcode (the highest-UMI contig) was retained and clones were aggregated by CDR3 amino-acid sequence, with the per-cell count used as the clone abundance, giving 23 peripheral-blood repertoires (10 discovery and 13 validation libraries; 86,394 cells; 55,678 productive TRB clones). These were passed through the same diversity routine used for the tumour cohorts, the same exact-CDR3 VDJdb annotation, and the same exact-CDR3 and CDR3 k-mer cross-compartment sharing analysis. Tumour-versus-cardiac compartment metrics were compared with two-sided Mann-Whitney U tests and Benjamini-Hochberg FDR correction (significance threshold FDR < 0.05); patient-level cardiac sub-group contrasts that did not reach this threshold are reported descriptively only.

## Results

### A reproducible pipeline for TCR repertoire reconstruction from public thoracic-tumour RNA-seq

We assembled an open-source pipeline (**Figure 1**; **Supplementary Figure S1**). The pipeline begins with an ENA/SRA query for paired-end Illumina bulk RNA-seq runs from four thoracic-tumour cohorts: GSE126044 and GSE135222 (NSCLC anti–PD-1 nivolumab), GSE145370 (ESCC paired chemo) and GSE193258 (NSCLC neoadjuvant pembrolizumab). The query returned 121 RNA-seq runs and approximately 1 TB of fastq. After de-prioritising under-represented runs and capping each cohort at 25 samples for the pilot, 25 runs entered the assembly stage. Reads were aligned and re-assembled with TRUST4 v1.1.5-r573 (26) against the IMGT-augmented BCR/TCR reference; only contigs reported as productive TRBV–CDR3–TRBJ rearrangements were retained. Diversity, clonality, V/J usage, CDR3 motif and VDJdb antigen-recognition modules were then applied per sample (**Supplementary Figure S1**). All software versions are listed in **Supplementary Table 5**.

The pipeline retained 12 productive repertoires from 3 cohorts (GSE126044 n = 6; GSE135222 n = 5; GSE145370 n = 1; **Figure 1A**) after two sequential exclusion steps documented in **Supplementary Figure S8**: thirteen samples failed download or gzip integrity (**Supplementary Tables 1**, **4**) and one cohort failed assembly-level QC (next section). The retained atlas contains 916 productive TRB clones across the three cohorts (atlas_summary.json), with raw productive-TRB counts spanning 1 (the lower bound) to 526 per sample (**Figure 1B**; **Supplementary Tables 1**, **2**).

### Cohort-level assembly QC excludes a library-prep–incompatible cohort

The five GSE193258 samples that entered TRUST4 yielded zero productive TRB clones despite TRUST4 reporting tens of thousands of candidate reads, prompting a contig-level diagnostic (**Supplementary Figure S4**; **Supplementary Table 6**). On two cohort-representative samples, only 0.0% and 5.3% of TRUST4 assemblies spanned both a V- and a J-gene (V+J). The two control samples drawn from the retained cohorts reached 83.1% and 70.5% V+J (**Supplementary Figure S4** right panels). GSE193258 assemblies also showed elongated CDR3-nucleotide lengths (median 43.5–45.0 nt versus 36.0–39.0 nt in V+J-rich references; **Supplementary Figure S4** centre panel), consistent with V-only assemblies in which truncated CDR3-encoding reads accumulate without a closing J-segment. We therefore defined a cohort-level QC rule (≥ 10% V+J assemblies on ≥ 1 representative sample) and excluded the entire GSE193258 cohort before any downstream comparison (**Supplementary Figure S8**).

Among the retained 12 samples, productive-TRB counts correlated tightly with TRUST4-mapped reads (Spearman ρ = 0.944; Pearson r on log10 = 0.977 over n = 12; **Supplementary Figures S3A, B**). The log10–log10 slope of 0.90 (GSE126044) and 0.87 (GSE135222) indicates sub-linear clone accumulation with sequencing depth, as expected from rarefaction theory. Residual analysis (**Supplementary Figures S3C, D**) showed no outlier sample with disproportionate clone gain or loss after depth adjustment.

### Diversity and clonality landscape of the retained pilot dataset

Per-sample diversity in NSCLC anti–PD-1 cohorts ranged from highly skewed (Shannon H ≈ 1.05; SRR8526725) to near-uniform (Shannon H ≈ 4.21; SRR9879275; **Figures 1C**, **2A**; **Supplementary Table 2**). The single ESCC sample (SRR11094249, 526 clones) (n = 1 after QC; all ESCC observations reported here therefore derive from this single sample and should be read as illustrative) sat at intermediate entropy (H = 3.18) but reached the highest clonality of the atlas (1 − H/log S = 0.49) (clonality = 1 − Shannon H/log S, where higher values indicate a more oligoclonal, dominated repertoire), driven by a single dominant clone (**Figure 1D**). Inverse-Simpson effective species spanned 2.78–35.13, with NSCLC cohorts overlapping (**Figure 2B**). Hill-number profiles (q = 0, 1, 2) sloped downward from richness to dominance-weighted estimators, with ESCC showing the steepest collapse from q = 0 to q = 2 (S0 = 526 → S2 = 4.81), confirming an oligoclonal architecture (**Figure 1E**; **Supplementary Figure S5**). At q = 0, NSCLC nivolumab samples covered 3–113 unique TRBs (**Supplementary Figure S5** bottom-right).

**Figure 6:**
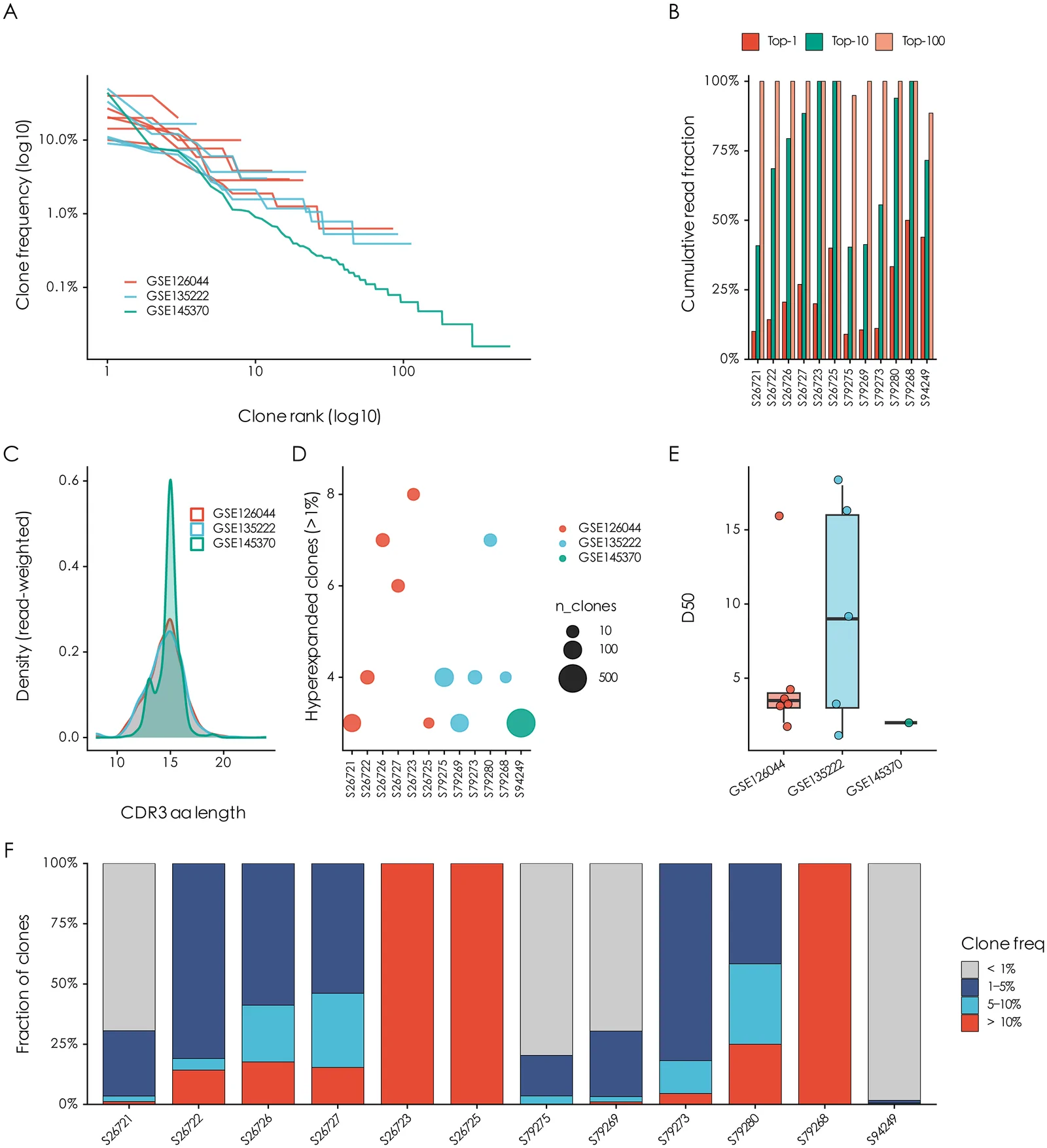
Clonal expansion and CDR3-length distribution. **(A)** Rank–frequency log–log plots of per-sample clone frequencies, coloured by cohort. **(B)** Cumulative read fraction captured by the top-1/top-10/top-100 clones per sample, displayed as stacked bars. **(C)** Read-weighted CDR3-amino-acid length density distributions per cohort. **(D)** Number of clones with read frequency > 1% per sample; symbol size proportional to total productive clones in that sample. **(E)** D50 by cohort (mirror of **Figure 1F** shown alongside the other clone-expansion panels). **(F)** Per-sample stacked bar of clone fractions by read-frequency bin (< 1%, 1–5%, 5–10%, > 10%).

D50 (the number of clones contributing the top 50% of reads) ranged from 1 to 18 in NSCLC samples and was 2 in the ESCC sample (**Figures 1F**, **6E**). D50 was uncorrelated with raw clone counts on this scale: SRR9879275 carried 113 clones at D50 = 18, while SRR11094249 carried 526 clones at D50 = 2. Pielou evenness, in contrast, was high across NSCLC samples (J ≥ 0.86; **Figure 2D**) and dropped to J = 0.51 in ESCC, consistent with the Gini coefficient pattern (NSCLC 0.13–0.46 versus ESCC 0.86; **Figure 1G**). Chao1 richness scaled with observed clones on a log–log slope close to unity (**Figure 2E**), suggesting the rare-clone tail was still under-sampled at current depth. Rarefaction curves corroborated this: all 11 NSCLC samples and the ESCC sample showed unique-clone counts continuing to rise from 50% to 100% subsampling (**Supplementary Figures S6A, C**). The Chao1/observed ratio remained > 1.0 for every sample (range 1.00–7.48; **Supplementary Figure S6B**), and the fraction of unique clones recovered at 50% depth was 0.55–0.71 (**Supplementary Figure S6E**). The pilot therefore sits well below the rarefaction asymptote.

### Differential V/J usage and clonal skewing across cohorts

V-gene usage was visualised as per-sample TRBV frequency heatmaps for the two larger cohorts (**Figures 3A, B**), with TRBV × TRBJ pair counts in **Figure 3F**. Aggregated V-gene rank curves (**Figure 3C**) showed an ESCC-driven tail dominated by a single TRBV28 peak with mean per-sample count ~ 3,000, an order of magnitude above any NSCLC TRBV. The single most-used TRBV–TRBJ pair across the atlas was TRBV28–TRBJ1–5 with 2,794 reads (**Figure 3E**; **Supplementary Table 7**), entirely contributed by the ESCC sample. Per-sample V-gene entropy (Shannon over TRBV) was 2.0–3.0 for NSCLC samples and 2.0 for ESCC (**Figure 3D**), confirming that the ESCC repertoire concentrates reads but not gene families. Cohort-paired V-gene preference plots (**Figures 3G, H**) ordered TRBV genes by log2 cohort-vs-other fold-change, recovering TRBV12-2, TRBV20-1, TRBV24-1, TRBV12–4 and TRBV3–2 among the cohort-distinctive segments.

CDR3 amino-acid length distributions were unimodal and centred at 14–15 aa in all three cohorts (**Supplementary Figures S2A–C**). The cross-cohort read-weighted overlay showed tighter peaks in ESCC, reflecting the dominant clone (**Supplementary Figure S2D**; **Figure 6C**). No cohort showed a systematic CDR3-length shift larger than 1 aa at the mode.

### Hyperexpanded clones dominate the ESCC repertoire

We stratified clones into low (< 1%), medium (1–5%), high (5–10%) and hyperexpanded (> 10%) read-frequency bins per sample (**Figure 6F**). All six GSE126044 NSCLC samples and four of five GSE135222 NSCLC samples were dominated by the < 1% bin (light grey, **Figure 6F**), with at most one clone exceeding 10%. SRR9879280 and SRR9879268 (both GSE135222) contained ≥ 1 hyperexpanded clone (orange band > 10%). The ESCC sample (SRR11094249) was qualitatively different: a single CDR3 (CASTTSFQGYQPQHF, TRBV28–TRBJ1-5) accounted for 42.6% of all productive reads, with the next two clones at 7.5% and 6.9% (**Supplementary Table 7**). Clone-size rank–frequency plots (**Figure 6A**) showed log–log slopes that decoupled the ESCC sample from the NSCLC cohorts. The cumulative read fraction captured by the top-1, top-10 and top-100 clones (**Figure 6B**) reached 100% at top-100 for nine of twelve samples, a direct consequence of the pilot-scale productive-clone counts. The number of clones exceeding 1% read frequency per sample (**Figure 6D**) ranged from 3 to 8 across the atlas.

### VDJdb-annotated TCRs span viral memory and tumour/self-antigens

Exact CDR3-amino-acid matches to VDJdb returned 37 antigen hits across 11 of the 12 productive samples (**Figure 5A**; **Supplementary Table 3**). The hit yield was disproportionate by cohort: 6 hits in GSE126044, 11 in GSE135222 and 20 in GSE145370 (**Figure 5C**). Hits scaled with productive clone count (**Figure 5F**), with the single ESCC sample contributing 20 of 37 hits. By species, the recovered TCRs were dominated by human cytomegalovirus (CMV) and influenza A, with additional hits to EBV, HIV-1, MCPyV, MCMV, SARS-CoV-2 and Homo sapiens self-antigens (**Figure 5B**; **Supplementary Figure S7** left panels). Most hits were MHC-I-restricted (33/37), with four MHC-II–restricted TCRs reactive to HIV-1 Gag (DRFYKTLRAEQASQEV), insulin VEALYLVCG and EBV PKYVKQNTLKLAT epitopes (**Figure 5C**; **Supplementary Figure S7** centre-left).

Stratifying hits by epitope category (**Figure 5E**), four of 37 hits across the atlas were tumour- or self-antigen reactive. These comprised a NY-ESO-1 TCR (CASSLVGLAGELFF, SLLMWITQV epitope) recovered from GSE126044 (SRR8526726) and again from GSE145370 (SRR11094249), an MLANA/MART-1 ELAGIGILTV-binding TCR (CASSSTGVGQPQHF), and an MCPyV T-Ag AAFKRSCLK-binding TCR (CASSQDPGSSYNEQFF). The remaining 33 hits were viral-memory TCRs, with the top-ranked individual epitopes being influenza A matrix protein M1 GILGFVFTL (7 hits), CMV pp65 NLVPMVATV (6 hits) and CMV IE1 KLGGALQAK (4 hits) (**Supplementary Figure S7** right). Per-sample top-hit clones were of mid-rank within their repertoires (**Figure 5G**), with annotated frequencies between 0.05% and 10%. That most annotated clones were of mid-repertoire rank rather than dominant is consistent with a bystander virus-specific memory population rather than antigen-driven dominant expansion.

### Cohort-unique CDR3 k-mer motifs with no cross-cohort clone sharing

Pairwise inter-cohort Jaccard similarity over full CDR3-amino-acid clonotypes was 0.000 for every cohort pair (**Figure 4A**). The cumulative-sharing analysis (**Figure 4D**) confirmed that all 916 clones were carried by exactly one cohort; no clone appeared in two cohorts. (cross-cohort sharing was assessed on the full CDR3-amino-acid + V + J clonotype identifier; an identical CDR3-amino-acid sequence carrying a different V/J call — such as the NY-ESO-1-annotated CASSLVGLAGELFF recovered in two cohorts — is therefore not counted as a shared clone) We therefore decomposed CDR3 sequences into 3-mer, 4-mer and 5-mer fragments and asked whether short motifs were shared. The top k-mers per length all corresponded to TCR-scaffold residues (k = 3: CAS, ASS, CSA, GEL, TGE, LFF, SSL, EQF, QFF; k = 4: CASS, TGEL, GELF, NEQF, etc.; **Figure 4B**) and were detected in every sample, reflecting universal CDR3-beta architecture rather than antigen-driven convergence. Cohort-level k-mer overlap (**Figure 4C**) was 100% at k = 3 for every cohort pair. The overlap dropped to 24–32% at k = 4 and was negligible at k = 5 for every cross-cohort pair, with diagonal cells fixed at 100% by definition.

Counting cohort-unique k = 4 motifs (**Figure 4E**), GSE126044 contributed 587 unique 4-mers out of 1,029 detected, GSE135222 contributed 940 of 1,570, and GSE145370 (the single ESCC sample) contributed 2,211 of 2,947. The larger ESCC pool reflects the higher productive-clone count rather than greater per-clone diversity. The pilot atlas therefore shows no clone-level cross-cohort overlap and only scaffold-level k-mer sharing.

### Extending the atlas to ICI cardiac immunotoxicity

Applying the identical pipeline to 23 peripheral-blood ICI-irAE repertoires (**Supplementary Table 8**) placed the cardiac ICI-irAE cohort (GSE180045; discovery and validation batches) alongside the three tumour cohorts in the atlas landscape (**Figures 1H, I**; descriptive inventory only). The single statistically significant cross-compartment contrast was clonal structure: thoracic-tumour tissue repertoires were markedly more oligoclonal than the peripheral cardiac repertoires (top-10 clone fraction 0.76 vs 0.18; top-1 fraction 0.20 vs 0.05; inverse Simpson 8.8 vs 177; Shannon 2.7 vs 6.5; observed clones 19 vs 1,897; D50 3.5 vs 221; Mann-Whitney with Benjamini-Hochberg FDR, all seven depth-sensitive metrics FDR < 0.001; **Figure 2F** left). This contrast must be read with an explicit depth caveat: the cardiac libraries are 10x single-cell T-cell-enriched (thousands of clones per library) whereas the tumour libraries are bulk RNA-seq (4–526 TCRs per library), so the difference is partly a sequencing-depth and compartment (tissue versus peripheral blood) effect rather than a purely biological one. Consistent with this, depth-normalised, depth-independent metrics (clonality, Pielou evenness, Gini) did not differ between compartments (FDR = 0.61, n.s.; **Figure 2F** right) — once depth is accounted for, the compartment difference disappears.

All within-patient cardiac contrasts (myocarditis versus no-irAE; myocarditis-containing versus non-myocarditis; any irAE versus no-irAE) were not statistically significant in this small single-cell cohort (each group n = 3-4; FDR > 0.6 or P ≥ 0.2) and are therefore reported descriptively only. Likewise, exact-CDR3 VDJdb annotation of the cardiac clones returned 1,980 hits dominated by viral memory (72% viral, 22% self or tumour, 6% other), and 27 exact CDR3 amino-acid sequences were shared between the tumour and cardiac compartments; these were mostly short, public or convergent sequences whose motif overlap was confined to the TCR scaffold, consistent with the tumour-only analysis. Neither the antigen composition nor the shared-clone count differed significantly between compartments (Fisher P = 0.11 and P = 0.089), so we present them as hypothesis-generating observations that require prospective, paired myocardial-tumour TCR validation rather than as evidence of antigen mimicry.

## Discussion

We assembled a reproducible, openly-released pipeline that recovers productive TRB repertoires from public bulk RNA-seq of thoracic tumours and applied it to four GEO cohorts. After fastq integrity QC and a new cohort-level V+J-assembly QC rule, three cohorts and twelve productive samples were retained, yielding 916 productive TRB clones and 37 VDJdb-annotated antigen hits. The retained atlas separates the ESCC sample from the NSCLC anti-PD-1 samples on diversity, clonality, Gini coefficient and V-gene usage. It also recovers TCRs spanning viral memory (CMV, influenza A, EBV, SARS-CoV-2, MCMV, HIV-1) and tumour or self-antigens (NY-ESO-1, MART-1, MCPyV T-Ag, insulin). No clone was shared across cohorts, and k-mer overlap was confined to TCR-scaffold residues. We emphasise that this is a proof-of-concept pilot resource, and that all cross-cohort observations are descriptive and hypothesis-generating rather than statistically generalisable.

Our diversity and clonality estimates fall within the ranges reported by direct TCR-sequencing studies of solid tumours and ICI cohorts (5–8). Riaz and colleagues paired TCR-seq with RNA-seq in melanoma nivolumab patients (5), and Hopkins and colleagues used TCR-seq in pancreatic-cancer ICI patients (8). Our Shannon-entropy and clonality values for the retained NSCLC samples sit between those benchmarks. The recent benchmark by Peng and colleagues (28) shows that RNA-seq-based TCR inference recovers a sensitive but truncated repertoire. Only the more abundantly transcribed clones cross the detection floor, and clonality estimates are biased toward dominance. Our rarefaction curves (**Supplementary Figure S6**) and Chao1/observed ratios (range 1.00–7.48) corroborate that interpretation directly. The rare-clone tail is under-sampled here, so any biological inference must remain at the level of dominance and antigen annotation rather than full-repertoire reconstruction. Single-cell-TCR atlases of pan-cancer T cells (40) and ESCC ecosystems (42, 43) reach much higher resolution per sample. The bulk-RNA-seq path we follow gives access to the larger, already-deposited inventory of thoracic cohorts that single-cell studies do not cover.

The antigen-hit pattern is consistent with the bystander-T-cell model of solid-tumour CD8 repertoires (36–39). Simoni and colleagues (36) and Scheper and colleagues (37) both showed that most CD8 T cells in human tumours, including lung tumours, do not recognise tumour-derived antigens. Many are virus-specific memory cells that have trafficked into or reside within the tumour bed. Rosato and colleagues (38) and Erkes and colleagues (39) extended this to demonstrate persistent function of these virus-specific cells. In our atlas, 33 of 37 VDJdb hits map to viral antigens. The top three single epitopes are influenza A matrix protein M1 (GILGFVFTL), CMV pp65 (NLVPMVATV) and CMV IE1 (KLGGALQAK), mirroring public-TCR analyses of human peripheral and tumour-infiltrating CD8 repertoires. Four hits map to recognised tumour or self-antigens (NY-ESO-1 SLLMWITQV, MART-1 ELAGIGILTV, MCPyV T-Ag AAFKRSCLK, insulin VEALYLVCG). NY-ESO-1 (57) and MART-1 (58) are canonical adoptive-TCR-therapy targets, and MCPyV T-Ag (59) is a viral oncoprotein recognised in Merkel cell carcinoma. The framework outlined by Schumacher and Schreiber (60) anticipates that neoantigen-specific TCRs are sparse, private and unlikely to be matched by exact-string lookup in any current public database. The four annotated tumour-antigen hits should therefore be read as a lower bound, and motif-aware matching (54–56) is a clear next step. We emphasise that exact CDR3-β string matches to VDJdb are database-level annotations consistent with, but not proof of, the listed antigen specificities, since identical CDR3-β sequences can arise convergently and may differ in Vα/Vβ pairing and HLA restriction.

The cohort-level QC rule that excluded GSE193258 is, to our knowledge, an early, explicitly stated and sample-checkable assembly-quality criterion (≥ 10% V+J assemblies on ≥ 1 representative sample) for RNA-seq-based TCR inference. Bolotin and colleagues drew attention to library-prep-dependent biases in TCR profiling (20, 27), and several BCR/TCR-assembly tools (22, 24, 25) have documented heterogeneous sensitivities. What has been missing is a public, sample-level checkable rule that can be applied before atlas integration. The diagnostic patterns in GSE193258 (V-only assemblies at 78.9–80.0% of contigs, and elongated CDR3-nucleotide lengths in those V-only contigs; **Supplementary Figure S4**) point to a 3′-biased or polyA-tagged RNA-seq chemistry on that cohort that under-recovers J segments. The same diagnostic can transfer to other immune-receptor inference workflows that depend on V- and J-spanning reads. We acknowledge that the 10% cutoff is a pragmatic heuristic rather than a calibrated optimum, and that the exact value should be refined as additional cohorts accumulate.

Our study has clear limitations. First, the retained atlas contains 12 productive samples from 3 cohorts, with one cohort contributing a single sample after QC. (the ESCC arm is represented by n = 1, so the ESCC-versus-NSCLC contrast should be read as illustrative) Cross-cohort comparisons are therefore descriptive rather than statistical, and no clinical-outcome modelling is attempted in this release. Second, the planned scope of five thoracic-tumour types (LUAD, LUSC, ESCA, MESO, THYM) reduces to two (NSCLC and ESCC) because the remaining cohorts’ primary RNA-seq is controlled-access via dbGaP. Third, antigen-recognition assessment uses exact CDR3-amino-acid matching to VDJdb only. McPAS-TCR (30) and IEDB (31) lookup modules are scaffolded but not run in this release. Structurally-similar near-neighbour matches via GLIPH2 (55) and tcrdist3 (56) are not counted, so the 37 reported hits should be read as a lower bound. Fourth, HLA typing scripts using arcasHLA (33) are released but not executed, so no MHC-restriction filtering of VDJdb hits was applied beyond the database’s own MHC-class annotations. Fifth, rarefaction analysis indicates that the rare-clone tail is under-sampled for every retained sample at present sequencing depth, which constrains any interpretation of richness-based metrics. The pipeline as released is positioned as a resource that addresses these limitations cohort-by-cohort. Three concrete extensions are immediate: adding ESCC neoadjuvant chemo-immunotherapy series (11), running arcasHLA (33), and routing each sample’s V/J-trimmed contigs through GLIPH2 (55) and tcrdist3 (56). These extensions will recover more biology and more antigen specificity from the same bulk inputs. The TCR-substrate scope can be extended to TRA and TRG via the same TRUST4 (26) call. The antigen layer can be re-built each time VDJdb (29), McPAS-TCR (30) or IEDB (31) issues a new release.

Finally, we extended the same pipeline to ICI-associated cardiac immunotoxicity by computationally re-mining a single-cell ICI-irAE cohort (18). Four points temper the interpretation. First, this is a computational re-mining, not new sampling. Second, the only statistically significant finding — greater oligoclonality of tumour tissue than of peripheral cardiac repertoires — is driven largely by sequencing depth and by tissue-versus-blood sampling: depth-normalised metrics (clonality, Pielou evenness, Gini) do not differ between compartments (**Figure 2F** right), so the contrast should not be read as an intrinsic difference in clonal architecture. Third, the patient-level cardiac sample size is small (3–4 patients per group); the within-cardiac comparisons, the antigen composition and the tumour-cardiac shared clones did not reach significance and are reported descriptively, pending prospective paired myocardial-tumour TCR studies. Fourth, the 27 tumour-cardiac shared CDR3 sequences are mostly short and public, and do not by themselves establish molecular mimicry. With these caveats, applying one uniform pipeline across both compartments offers a transparent starting point for studying the shared T-cell basis of anti-tumour immunity and ICI cardiotoxicity.

## Data Availability

Publicly available datasets were analyzed in this study. The cardiac ICI-irAE single-cell TCR data were obtained from the Gene Expression Omnibus under accession number GSE180045.

## Supplementary material

Supplementary Figure 1 End-to-end pipeline schematic. Block diagram of the six pipeline stages — ENA/SRA query (121 runs across 4 cohorts) → TRUST4 assembly (TRB + TRA, productive filter) → assembly QC → diversity and clonality → atlas integration → VDJdb/McPAS/IEDB antigen lookup → publication figures and tables. The table below the diagram lists the per-step rule, the live retention count and the outcome.

Supplementary Figure 2 CDR3-amino-acid length distributions per cohort. **(A)** GSE126044 (n = 147 clones across retained samples). **(B)** GSE135222 (n = 243 clones). **(C)** GSE145370 (n = 526 clones). **(D)** Read-weighted density overlay of all three cohorts on a common axis.

Supplementary Figure 3 Sequencing-depth versus productive-clone yield. **(A)** Productive TRB clones versus TRUST4-mapped reads per sample on a log–log scale; Spearman ρ = 0.944, Pearson r (log10) = 0.977, n = 12. **(B)** Cohort-split scatter with within-cohort regression lines. **(C)** Per-sample residuals (log10) from the cohort regression. **(D)** Per-cohort correlation coefficients (Pearson on log10 + Spearman). **(E)** log10–log10 slopes per cohort (GSE126044 slope = 0.90 over n = 6; GSE135222 slope = 0.87 over n = 5).

Supplementary Figure 4 GSE193258 exclusion evidence. (Top left) Per-sample fraction of TRUST4 assemblies by V+J/V-only/J-only call. (Top middle) CDR3-nucleotide-length density distributions for the four diagnostic samples (two good-cohort references, two GSE193258 problematic samples). (Top right) Per-sample V+J fraction (SRR8526721 83.1%; SRR11094249 70.5%; SRR17491517 5.3%; SRR17491496 0.0%). (Bottom left) Per-sample V-only fraction; SRR17491496 and SRR17491517 dominated by V-only assemblies. (Bottom right) Total TRUST4 assemblies per diagnostic sample. Footer states: 2/2 GSE193258 samples fail < 10% V+J; the cohort is removed before atlas integration.

Supplementary Figure 5 Hill-number profiles and per-q effective-species summaries. (Top left) Per-sample Hill-number trajectories from q = 0 to q = 2 on a log scale. (Top right) Cohort-level median ± IQR Hill profiles. (Bottom left) Hill ratio q = 1/q = 2 per sample (a per-sample dominance index). (Bottom right) Effective species at q = 0, 1, 2 by cohort.

Supplementary Figure 6 Rarefaction analysis. **(A)** Per-sample rarefaction curves of unique productive TRB clones against subsampling fraction (0–100%), coloured by cohort, on a log scale. **(B)** Per-sample Chao1/observed ratio. **(C)** Cohort-mean rarefaction curves with bootstrap range. **(D)** Per-sample unique clones detected at 50% subsampling. **(E)** Ratio of unique clones at 50% to clones at 100% per sample.

Supplementary Figure 7 VDJdb hit details. (Top left) Per-sample VDJdb-hit species composition (CMV, EBV, HIV-1, Homo sapiens, influenza A, MCMV, MCPyV, SARS-CoV-2). (Top middle) Per-sample VDJdb-hit MHC-class composition. (Top right) Top-10 individual epitopes by hit count (GILGFVFTL n = 7; NLVPMVATV n = 6; KLGGALQAK n = 4; SLLMWITQV n = 2; RAKFKQLL n = 2; IPSINVHHY n = 2; …). (Bottom left) MHC-II epitopes recovered (VEALYLVCG, PKYVKQNTLKLAT, DRFYKTLRAEQASQEV). (Bottom middle) Distribution of VDJdb hits per CDR3 clone (most clones with 1 hit, six with 2 hits). (Bottom right) Viral versus tumour/self-hit fraction per cohort.

Supplementary Figure 8 Atlas attrition flow chart. Sankey-style flow of samples from the original ENA query (121 runs, 4 cohorts) through atlas selection (25 runs after cohort cap and productivity rank), cohort-level QC (0 GSE193258 runs retained after < 6% V+J), download integrity QC (13 excluded for incomplete/corrupt files) and the productive-TRB filter, to the final atlas of 12 productive samples from 3 retained cohorts.

Supplementary Table 1 Atlas manifest with QC status. Per-sample cohort, library layout, instrument model, fastq R1/R2 download status, TRUST4 run status, productive-TRB clone count and exclusion reason for every sample evaluated.

Supplementary Table 2 Full diversity metrics dump. Per-sample observed clones, total reads, Shannon, Simpson, inverse Simpson, Pielou evenness, Gini, Chao1, Hill numbers (q = 0/1/2), top-1/top-10/top-100 read fractions, D50 and clonality, for the 12 productive samples.

Supplementary Table 3 Full VDJdb matches. Per-hit record (sample, CDR3-amino-acid sequence, number of VDJdb hits per CDR3, recovered epitopes, antigens, species, MHC class) for all 37 hits across 11 of 12 productive samples.

Supplementary Table 4 Excluded atlas samples with reasons. Per-sample exclusion category (fastq incomplete or missing; no productive TRB) and per-sample evidence note (R1/R2 status; downstream TRUST4 outcome).

Supplementary Table 5 Software versions used in the ThoracicTCR pipeline. Tool, version string and source for every executable and library used, including TRUST4, samtools, gdc-client, aria2c, arcasHLA, Python and Python packages, R and R packages, the OS kernel and the R interpreter.

Supplementary Table 6 GSE193258 exclusion evidence. Per-sample TRUST4 assembly counts split by V-only/J-only/V+J/none, percentage of V+J assemblies, median and mean CDR3-nucleotide length, and a one-line interpretation; reference samples from GSE126044 and GSE145370 included for direct comparison.

Supplementary Table 7 Top 20 expanded productive TRB clones with VDJdb annotation. Per-clone sample, cohort, CDR3-amino-acid sequence, V-call, J-call, read count, repertoire-level frequency and VDJdb annotation (epitope, antigen, species, MHC class) for the twenty most expanded productive clones in the atlas; the top entry (CASTTSFQGYQPQHF, TRBV28/TRBJ1-5; SRR11094249, GSE145370) carries 42.58% of all productive reads in its sample.

Supplementary Table 8 Cardiac (ICI-irAE) repertoire manifest and diversity metrics. Per-library batch (discovery or validation), ICI disease group, disease state, patient or pool identity, cell count, productive-TRB clone count and diversity metrics (Shannon, inverse Simpson, clonality, top-10 clone fraction and D50) for the 23 peripheral-blood cardiac repertoires (GSE180045; 10 discovery and 13 validation libraries) processed through the identical pipeline. The tumour-versus-cardiac compartment-comparison statistics are reported in the **Figure 2F** annotation.
